# Comparison of cardiac magnetic resonance imaging, functional and haemodynamic variables in pulmonary arterial hypertension: insights from REPAIR

**DOI:** 10.1183/23120541.00547-2023

**Published:** 2024-02-12

**Authors:** David G. Kiely, Richard Channick, Dayana Flores, Nazzareno Galiè, Gwen MacDonald, J. Tim Marcus, Lada Mitchell, Andrew Peacock, Stephan Rosenkranz, Ahmed Tawakol, Adam Torbicki, Anton Vonk Noordegraaf, Andrew J. Swift

**Affiliations:** 1 Sheffield Pulmonary Vascular Disease Unit and NIHR Biomedical Research Centre, Royal Hallamshire Hospital and University of Sheffield, Sheffield, UK; 2 Department of Clinical Medicine, University of Sheffield, Sheffield, UK; 3 David Geffen School of Medicine at UCLA, Los Angeles, CA, USA; 4 Global Medical Affairs, Actelion Pharmaceuticals Ltd, a Janssen Pharmaceutical Company of Johnson & Johnson, Allschwil, Switzerland; 5 Cardiology Unit, IRCCS Azienda Ospedaliero-Universitaria di Bologna, Bologna, Italy; 6 Dipartimento di Medicina Specialistica Diagnostica e Sperimentale (DIMES), Università di Bologna, Bologna, Italy; 7 Amsterdam UMC, Vrije Universiteit Amsterdam, Amsterdam, The Netherlands; 8 Statistical Decision Science, Actelion Pharmaceuticals Ltd, a Janssen Pharmaceutical Company of Johnson & Johnson, Allschwil, Switzerland; 9 Scottish Pulmonary Vascular Unit, Glasgow, UK; 10 Department of Cardiology, Heart Center, University Hospital Cologne and Cologne Cardiovascular Research Center, University of Cologne, Cologne, Germany; 11 Cardiology Division, Massachusetts General Hospital and Harvard Medical School, Boston, MA, USA; 12 Department of Pulmonary Circulation, Thromboembolic Disease and Cardiology, Centre for Postgraduate Medical Education ECZ-Otwock, ERN-LUNG Member, Otwock, Poland

## Abstract

**Background:**

Measures that can detect large treatment effects are important for monitoring therapeutic effectiveness. The 2022 European Society of Cardiology/European Respiratory Society guidelines highlight the importance of imaging in monitoring disease status and treatment response in pulmonary arterial hypertension (PAH). Are the standardised treatment effect sizes (STES) of cardiac magnetic resonance imaging (cMRI) comparable with functional and haemodynamic variables?

**Methods:**

REPAIR (ClinicalTrials.gov: NCT02310672) was a prospective, multicentre, single-arm, open-label, 52-week phase 4 study evaluating the effect of macitentan 10 mg, with or without a phosphodiesterase 5 inhibitor (PDE5i), on right ventricular (RV) remodelling, cardiac function and cardiopulmonary haemodynamics. Both cMRI and functional assessments were performed at screening and at weeks 26 and 52; haemodynamic measurements were conducted at screening and week 26. In this *post hoc* analysis, STES were estimated using the parametric Cohen's d and non-parametric Cliff's delta tests.

**Results:**

At week 26, large STES (Cohen's d) were observed for 10 of the 20 cMRI variables assessed, including the prognostic measures of RV and left ventricular stroke volume and RV ejection fraction and the haemodynamic trial end-point, pulmonary vascular resistance; medium STES were observed for 6-min walk distance (6MWD). The STES were consistent in treatment-naïve patients and those escalating therapy and maintained at week 52. Similar results were obtained using the non-parametric Cliff's delta method.

**Conclusions:**

The treatment effect of macitentan, alone or in combination with a PDE5i, was comparable for several cMRI and haemodynamic variables with prognostic value in PAH, and greater than that of 6MWD in patients with PAH, highlighting the emerging relevance of cMRI in PAH.

## Shareable abstract

**These analyses highlight the potential of cMRI variables as a non-invasive way to monitor patients’ PAH disease status and predict clinically relevant outcomes, and support their further investigation as surrogate end-points in PAH trials** https://bit.ly/46YxQLd

## Introduction

Pulmonary arterial hypertension (PAH) is a progressive condition affecting the pulmonary vasculature resulting in elevated pulmonary vascular resistance (PVR) and pulmonary arterial pressure (PAP), eventually leading to right ventricular (RV) failure and death [1]. Despite significant improvements in outcomes following the development of effective therapies, PAH remains a life-shortening condition and new approaches to clinical research and management are needed.

Historically, clinical trials in PAH were single agent, placebo controlled, of short duration and comprised a relatively small population, employing functional assessments such as 6-min walk distance (6MWD) and right heart catheterisation (RHC) measurements such as PVR as end-points [2, 3]. While still used in early-phase studies [2, 3], the small treatment effect sizes observed for these short-term end-points in patients on background PAH therapies [2] has led to an evolution of trial design into larger studies using composite morbidity/mortality end-points [4–6]. The longer duration and large patient numbers needed make these trials challenging to conduct. Hence, surrogate end-points capable of identifying large treatment effect sizes over a short follow-up period, where the magnitude of change reflects clinically important long-term outcomes, are highly desirable.

The 2022 European Society of Cardiology/European Respiratory Society guidelines [7, 8] highlight the value of assessing the right ventricle using imaging techniques, including the gold standard of cardiac magnetic resonance imaging (cMRI) and echocardiography. Furthermore, a recent meta-analysis reinforced cMRI as a powerful prognostic marker in a large cohort of PAH patients [9], and several cMRI measures have been proven to add prognostic value during initial risk assessment, allowing for monitoring of disease status and treatment response at follow-up [10–12] with high repeatability [13].

A recent analysis reported the standardised treatment effect sizes (STES) of variables measured in PAH patients in the RESPIRE study by using Cohen's d statistic [13]. This converts the change in a variable affected by treatment to a unitless measure, permitting its direct comparison with other variables. In contrast to 6MWD and N-terminal pro-brain natriuretic peptide (NT-proBNP), only cMRI variables demonstrated large STES, including in patients receiving background PAH therapy [13]. Together, these data provide support for use of cMRI metrics in the assessment of disease status and treatment response in PAH; however, the RESPIRE study lacked RHC measurements.

In the prospective, open-label REPAIR study with macitentan (an oral endothelin receptor antagonist indicated for the long-term treatment of PAH) [14, 15], a decrease in PVR of 38% was observed after 26 weeks of treatment with macitentan (alone or in combination with a phosphodiesterase 5 inhibitor (PDE5i)) [16], in line with results from the pivotal macitentan SERAPHIN study [4]. An increase in RV stroke volume (RVSV) of 12.0 mL and improvements in the majority of RV and left ventricular (LV) variables measured were also observed, suggesting that PAH therapy contributes to beneficial remodelling of the right ventricle in PAH patients [16].

This *post hoc* analysis compared the STES of cMRI metrics in REPAIR to currently used PAH end-points (PVR and 6MWD [3]) and other haemodynamic and non-invasive functional measures including NT-proBNP.

## Methods

### REPAIR study design

REPAIR (ClinicalTrials.gov: NCT02310672) was a prospective, multicentre, single-arm, open-label, 52-week, phase 4 study (supplementary figure S1); the study design has been described previously [16]. Eligible patients were 18–74 years of age with idiopathic or heritable PAH, or PAH related to connective tissue disease, drug use or toxin exposure, or simple congenital systemic-to-pulmonary shunts at least 2 years after repair. Patients were required to have a 6MWD of ≥150 m, be in World Health Organization Functional Class (WHO FC) I–III and be PAH treatment-naïve or on a stable (≥3 months) PDE5i regimen at screening. For treatment-naïve patients, physicians had the option to initiate macitentan as monotherapy or as initial combination therapy with a PDE5i.

### Clinical assessments

cMRI and assessments of 6MWD, WHO FC and NT-proBNP were performed at screening and at weeks 26 and 52. RHC was performed at screening and at week 26.

### Statistical methodology

The safety set for the REPAIR study comprised all enrolled patients who received at least one dose of macitentan [16]. For these *post hoc* analyses, STES estimations were produced for continuous variables from data of all participants in the safety analysis set with both baseline and post-baseline (week 26 and/or 52) measurements. Additionally, data for two subgroups of the safety set are presented: 1) treatment-naïve patients (those who initiated macitentan as monotherapy or as initial combination therapy with a PDE5i) and 2) patients escalating therapy (those on stable background PDE5i for ≥3 months prior to study start).

### Estimates of treatment effect size

Cohen's d was chosen to estimate the STES of macitentan, alone or in combination with a PDE5i. It was computed as a ratio of the mean change from baseline to post-baseline measurements (week 26 or 52) and the standard deviation of that change (*i.e.* according to paired sample design methodology). Non-central *t* confidence limits (CL; 95%) for the paired sample design were also calculated [17]. The Cohen's d STES were categorised as: <0.20=no change, 0.20– <0.50=fair change, 0.50– <0.80=medium change and ≥0.80=large change, based on previously reported thresholds [18].

Cohen's d is a parametric estimator and is therefore susceptible to bias by outliers and skewed variable distributions. Given this, a supportive analysis was conducted to estimate the treatment effect sizes and corresponding 95% CLs using the non-parametric Cliff's delta approach [18]. In this method, a ratio is calculated from the number of patients with improvements minus the number of patients with worsening at follow-up (week 26 or 52) and the product of the number of patients with baseline and with post-baseline measurements. The Cliff's delta STES were categorised as: <0.11=no change, 0.11– <0.28=small change, 0.28– <0.43=medium change and ≥0.43=large change, based on previously reported thresholds [19].

Further details can be found in the supplementary material. All analyses were performed using SAS version 9.4 (SAS, Cary, NC, USA).

### Monitoring and ethics statement

Ethical approval was received from independent ethics committees/institutional review boards (supplementary appendix I) and the study was conducted in compliance with the Declaration of Helsinki. Written informed consent was obtained from all patients. cMRI results were assessed by a blinded central imaging committee.

## Results

### Analysis set and characteristics

A total of 112 patients were screened across 11 countries, with 87 patients receiving at least one dose of macitentan (safety set). Baseline characteristics are shown in table 1. The majority of patients were female (80.5%) and the median (range) age was 45 (19–74) years. The majority of patients (n=48 (55.2%)) had idiopathic PAH and were WHO FC II (46.0%) and III (52.9%) at baseline. The mean±sd 6MWD at baseline was 401.1±117.0 m. Most patients were treatment-naïve (n=56 (64.4%)): 22 (25.3%) initiated macitentan as monotherapy and 34 (39.1%) initiated macitentan as initial combination therapy with a PDE5i; 31 (35.6%) patients were escalating therapy. Median (interquartile range) macitentan exposure time was 52.1 (51.3–52.7) weeks in the safety set; four patients discontinued prior to the week 26 assessment.

**TABLE 1:** Demographics and baseline disease characteristics and therapy: safety set (n=87)

**Sex**	
Male	17 (19.5)
Female	70 (80.5)
**Age (years)**	45 (19–74)
**Age at PAH diagnosis (years)**	43 (18–73)
**BMI (kg·m^−2^)**	24.9±4.6
**PAH aetiology**	
Idiopathic PAH	48 (55.2)
Heritable PAH	4 (4.6)
Drug and toxin-induced	3 (3.4)
PAH associated with congenital heart disease^#^	5 (5.7)
PAH associated with connective tissue disease	27 (31.0)
**6MWD (m)**	401.1±117.0
**WHO FC at baseline**	
I	1 (1.1)
II	40 (46.0)
III	46 (52.9)
**Treatment strategy**	
Naïve patients initiating therapy	56 (64.4)
Macitentan initiated as monotherapy	22 (25.3)
Macitentan initiated as initial combination therapy with a PDE5i	34 (39.1)
Patients escalating therapy (*i.e.* initiating macitentan on top of stable background PDE5i)	31 (35.6)

Data are presented as n (%), median (range) or mean±sd. PAH: pulmonary arterial hypertension; BMI: body mass index; 6MWD: 6-min walk distance; WHO FC: World Health Organization Functional Class; PDE5i: phosphodiesterase 5 inhibitor. ^#^: only simple congenital systemic-to-pulmonary shunts at least 2 years post-surgical repair.

### Analysis of STES by Cohen's d statistic

**FIGURE 1:**
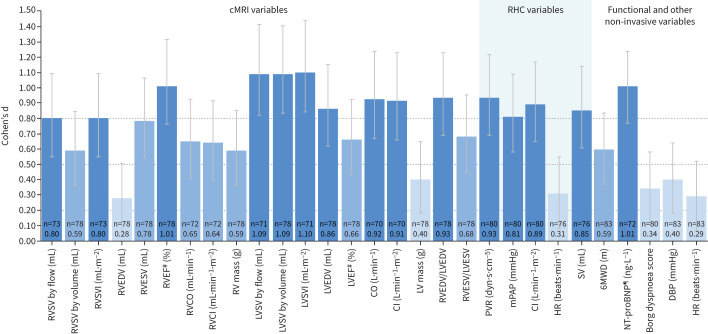
Cohen's d statistics for selected cardiac magnetic resonance imaging (cMRI), right heart catheterisation (RHC) and functional and other non-invasive variables at week 26 (safety set). Variables with no treatment effect are not included. ^#^: determined from standard volumetric measurements; ^¶^: log-transformed. n: number of patients with baseline and post-baseline measurements for a specific variable; Cohen's d statistics are reported below the n-values. For the standardised treatment effect sizes (STES): dark blue indicates a large change, mid-blue indicates a medium change and light blue indicates a small/fair change. The dotted lines at 0.20, 0.50 and 0.80 indicate the thresholds for a fair, medium or large change in STES, respectively. RV: right ventricular; RVSV: RV stroke volume; RVSVI: RVSV index; RVEDV: RV end-diastolic volume; RVESV: RV end-systolic volume; RVEF: RV ejection fraction; RVCO: RV cardiac output; RVCI: RV cardiac index; LV: left ventricular; LVSV: LV stroke volume; LVSVI: LVSV index; LVEDV: LV end-diastolic volume; LVEF: LV ejection fraction; CO: cardiac output; CI: cardiac index; LVESV: LV end-systolic volume; PVR: pulmonary vascular resistance; mPAP: mean pulmonary arterial pressure; HR: heart rate; SV: stroke volume; 6MWD: 6-min walk distance; NT-proBNP: N-terminal pro-brain natriuretic peptide; DBP: diastolic blood pressure.

**FIGURE 2:**
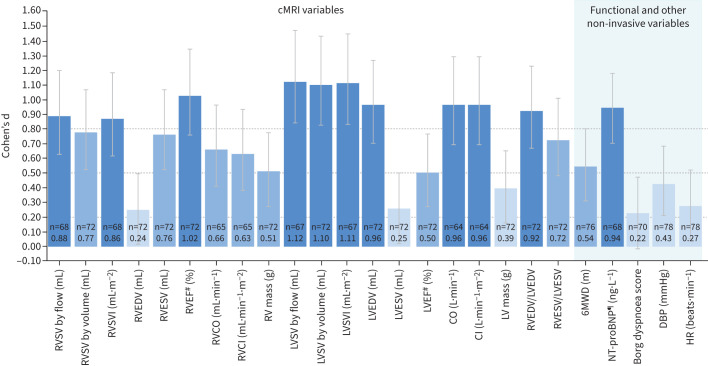
Cohen's d statistics for selected cardiac magnetic resonance imaging (cMRI), functional and other non-invasive variables at week 52 (safety set). Variables with no treatment effect are not included. ^#^: determined from standard volumetric measurements; ^¶^: log-transformed. n: number of patients with baseline and post-baseline measurements for a specific variable; Cohen's d statistics are reported below the n-values. For the standardised treatment effect sizes (STES): dark blue indicates a large change, mid-blue indicates a medium change and light blue indicates a small/fair change. The dotted lines at 0.20, 0.50 and 0.80 indicate the thresholds for a fair, medium or large change in STES, respectively. RV: right ventricular; RVSV: RV stroke volume; RVSVI: RVSV index; RVEDV: RV end-diastolic volume; RVESV: RV end-systolic volume; RVEF: RV ejection fraction; RVCO: RV cardiac output; RVCI: RV cardiac index; LV: left ventricular; LVSV: LV stroke volume; LVSVI: LVSV index; LVEDV: LV end-diastolic volume; LVESV: LV end-systolic volume; LVEF: LV ejection fraction; CO: cardiac output; CI: cardiac index; 6MWD: 6-min walk distance; NT-proBNP: N-terminal pro-brain natriuretic peptide; DBP: diastolic blood pressure; HR: heart rate.

The Cohen's d estimates of macitentan STES for cMRI, haemodynamic, functional and other non-invasive variables at weeks 26 and 52 are shown in tables 2 and 3 and figures 1 and 2. At week 26, large macitentan STES (Cohen's d ≥0.80) were observed for 10 of the 20 cMRI variables assessed: RVSV by flow, RVSV index (RVSVI), RV ejection fraction (RVEF), RV end-diastolic volume/LV end-diastolic volume (RVEDV/LVEDV) ratio, LV stroke volume (LVSV) measured by pulmonary artery flow and by volume, LVSV index (LVSVI), LVEDV, cardiac output and cardiac index. Of these, only LVSV measured by pulmonary artery flow and by volume and LVSVI had a 95% CL lower limit that remained above the 0.80 threshold. Large STES were also observed at week 26 for four of the six RHC variables assessed (PVR, mean PAP (mPAP), cardiac index and stroke volume). None of the functional and other non-invasive variables reported in the study had large STES as estimated by Cohen's d, except for log-transformed NT-proBNP (table 2 and figure 1 and supplementary figure S2a). Moderate STES (Cohen's d 0.50– <0.80) were observed at week 26 for the following seven cMRI variables assessed: RVSV by volume, RV end-systolic volume (RVESV), RV cardiac output, RV cardiac index, RV mass, RV end-systolic volume/LV end-systolic volume (RVESV/LVESV) ratio and LV ejection fraction, and also for 6MWD. Fair STES (Cohen's d 0.20– <0.50) were observed at week 26 for cMRI-assessed RVEDV, LV mass and RHC-assessed heart rate, and for the non-invasive variables of absolute NT-proBNP, Borg dyspnoea score, diastolic blood pressure and heart rate. At week 26, no change (Cohen's d <0.20) was observed for LVESV, mean right atrial pressure and systolic blood pressure.

**TABLE 2:** Change from baseline to week 26 and Cohen's d and Cliff's delta statistics for cardiac magnetic resonance imaging (cMRI), right heart catheterisation (RHC) and functional and other non-invasive variables: safety set (n=87)

	**n**	**Baseline**	**Week 26**	**Change from baseline** **to week 26**	**Cohen's d** **(95% CL)**	**Cliff's delta** **(95% CL)**
**cMRI variables**						
RVSV by flow (mL)	73	52.1±17.8	64.5±19.4	12.3±15.4	0.80 (0.55–1.09)	0.35 (0.20–0.49)
RVSV by volume (mL)	78	58.5±20.7	68.8±23.7	10.3±17.6	0.59 (0.36–0.85)	0.28 (0.12–0.42)
RVSVI (mL·m^−2^)	73	30.0±9.4	37.2±9.8	7.2±9.0	0.80 (0.55–1.09)	0.45 (0.29–0.58)
RVEDV (mL)	78	148.4±47.8	140.6±48.6	−7.8±28.4	0.28 (0.05–0.51)	0.12 (−0.04–0.27)
RVESV (mL)	78	90.0±40.9	71.8±34.1	−18.2±23.3	0.78 (0.54–1.06)	0.28 (0.12–0.42)
RVEF^#^ (%)	78	41.2±12.8	50.3±11.1	9.1±9.0	1.01 (0.76–1.32)	0.40 (0.26–0.53)
RVCO^¶^ (mL·min^−1^)	72	3.8±1.3	4.5±1.5	0.7±1.1	0.65 (0.41–0.92)	0.30 (0.14–0.45)
RVCI^¶^ (mL·min^−1^·m^−2^)	72	2.2±0.7	2.6±0.8	0.4±0.6	0.64 (0.40–0.92)	0.32 (0.16–0.46)
RV mass (g)	78	110.6±46.8	99.4±41.4	−11.3±19.1	0.59 (0.36–0.85)	0.14 (−0.02–0.29)
LVSV by flow (mL)	71	47.2±14.8	62.1±19.5	14.9±13.8	1.09 (0.82–1.42)	0.47 (0.31–0.60)
LVSV by volume (mL)	78	54.2±17.6	71.0±22.9	16.8±15.4	1.09 (0.83–1.40)	0.47 (0.32–0.60)
LVSVI (mL·m^−2^)	71	27.3±7.6	36.0±9.8	8.7±7.9	1.10 (0.84–1.44)	0.54 (0.38–0.66)
LVEDV (mL)	78	86.1±28.2	104.5±35.7	18.4±21.5	0.86 (0.62–1.15)	0.35 (0.20–0.48)
LVESV (mL)	78	31.9±16.0	33.6±17.7	1.7±10.9	NTE	NTE
LVEF^#^ (%)	78	64.0±10.9	68.9±10.1	4.9±7.4	0.66 (0.43–0.93)	0.29 (0.13–0.43)
LVCO^+^ (L·min^−1^)	70	3.5±1.1	4.3±1.4	0.9±0.9	0.92 (0.67–1.24)	0.42 (0.25–0.56)
LVCI^+^ (L·min^−1^·m^−2^)	70	2.0±0.6	2.5±0.7	0.5±0.6	0.91 (0.66–1.23)	0.44 (0.28–0.58)
LV mass (g)	78	103.5±24.3	107.3±24.8	3.9±9.7	0.40 (0.18–0.65)	NTE
RVEDV/LVEDV	78	0.5±0.3	0.3±0.3	−0.3±0.3	0.93 (0.69–1.23)	0.45 (0.30–0.57)
RVESV/LVESV	78	1.1±0.5	0.8±0.4	−0.3±0.4	0.68 (0.45–0.95)	0.31 (0.16–0.45)
**RHC variables**						
PVR (dyn·s·cm^−5^)	80	984.7±669.1	608.5±451.0	−376.2±404.8	0.93 (0.69–1.22)	0.49 (0.32–0.62)
mPAP (mmHg)	80	53.5±15.2	44.9±16.2	−8.6±10.6	0.81 (0.58–1.09)	0.34 (0.16–0.49)
mRAP (mmHg)	79	6.8±4.2	6.1±3.7	−0.7±4.1	NTE	NTE
Cardiac index (L·min^−1^·m^−2^)	80	2.4±0.7	3.0±0.7	0.6±0.6	0.89 (0.65–1.17)	0.44 (0.27–0.59)
Heart rate (beats·min^−1^)	76	77.6±13.0	73.8±11.7	−3.7±12.2	0.31 (0.08–0.55)	0.20 (0.01–0.37)
Stroke volume (mL)	76	55.7±20.2	71.5±19.7	15.8±18.6	0.85 (0.61–1.14)	0.46 (0.29–0.61)
**Functional and other non-invasive variables**						
6MWD (m)	83	405.3±116.7	448.7±110.6	43.3±73.5	0.59 (0.37–0.84)	0.27 (0.12–0.41)
Absolute NT-proBNP (ng·L^−1^)	72	1172.8±1824.0	481.7±879.4	−691.1±1612.8	0.43 (0.20–0.69)	0.37 (0.22–0.51)
Log-transformed NT-proBNP (ng·L^−1^)	72	6.3±1.3	5.4±1.1	−0.9±0.9	1.01 (0.77–1.24)	0.37 (0.22–0.51)
Borg dyspnoea score	80	3.9±2.1	3.1±2.0	−0.8±2.3	0.34 (0.12–0.58)	0.18 (0.02–0.33)
DBP (mmHg)	83	73.8±11.5	69.9±11.0	−3.9±9.7	0.40 (0.18–0.64)	0.18 (0.03–0.33)
SBP (mmHg)	83	116.0±14.0	115.6±14.8	−0.3±14.7	NTE	NTE
Heart rate (beats·min^−1^)	83	80.1±12.9	76.2±13.2	−3.9±13.4	0.29 (0.07–0.52)	0.19 (0.03–0.33)

Baseline, week 26 and change from baseline to week 26 data are presented as mean±sd. ^#^: determined from standard volumetric measurements; ^¶^: determined from pulmonary artery flow; ^+^: determined from aortic flow. Due to rounding, in some cases the difference between week 26 and baseline values does not equate to the change in baseline value. For the standardised treatment effect sizes: dark blue indicates a large change, mid-blue indicates a medium change and light blue indicates a small/fair change; NTE indicates no treatment effect (Cohen's d <0.20; Cliff's delta <0.11). CL: confidence limit; RV: right ventricular; RVSV: RV stroke volume; RVSVI: RVSV index; RVEDV: RV end-diastolic volume; RVESV: RV end-systolic volume; RVEF: RV ejection fraction; RVCO: RV cardiac output; RVCI: RV cardiac index; LV: left ventricular; LVSV: LV stroke volume; LVSVI: LVSV index; LVEDV: LV end-diastolic volume; LVESV: LV end-systolic volume; LVEF: LV ejection fraction; LVCO: LV cardiac output; LVCI: LV cardiac index; RHC: right heart catheterisation; PVR: pulmonary vascular resistance; mPAP: mean pulmonary arterial pressure; mRAP: mean right arterial pressure; 6MWD: 6-min walk distance; NT-proBNP: N-terminal pro-brain natriuretic peptide; DBP: diastolic blood pressure; SBP: systolic blood pressure.

**TABLE 3:** Change from baseline to week 52 and Cohen's d and Cliff's delta statistics for cardiac magnetic resonance imaging (cMRI) and functional and other non-invasive variables: safety set (n=87)

	**n**	**Baseline**	**Week 52**	**Change from baseline** **to week 52**	**Cohen's d** **(95% CL)**	**Cliff's delta** **(95% CL)**
**cMRI variables**						
RVSV by flow (mL)	68	51.9±17.8	65.5±20.5	13.6±15.4	0.88 (0.62–1.20)	0.35 (0.20–0.49)
RVSV by volume (mL)	72	59.0±21.2	72.1±24.6	13.1±17.1	0.77 (0.52–1.06)	0.34 (0.18–0.48)
RVSVI (mL·m^−2^)	68	30.1±9.6	37.9±10.2	7.8±9.0	0.86 (0.61–1.18)	0.43 (0.28–0.57)
RVEDV (mL)	72	148.0±46.2	141.2±45.5	−6.8±28.0	0.24 (0.01–0.49)	NTE
RVESV (mL)	72	89.0±38.4	69.1±31.5	−20.0±26.1	0.76 (0.52–1.06)	0.31 (0.16–0.45)
RVEF^#^ (%)	72	41.5±12.6	52.3±11.5	10.8±10.6	1.02 (0.76–1.34)	0.47 (0.32–0.59)
RVCO^¶^ (mL·min^−1^)	65	3.8±1.3	4.6±1.6	0.8±1.2	0.66 (0.41–0.96)	0.30 (0.13–0.44)
RVCI^¶^ (mL·min^−1^·m^−2^)	65	2.2±0.7	2.6±0.9	0.4±0.7	0.63 (0.38–0.93)	0.31 (0.15–0.46)
RV mass (g)	72	111.2±47.8	101.5±45.3	−9.7±19.0	0.51 (0.27–0.77)	0.12 (−0.04–0.28)
LVSV by flow (mL)	67	47.5±15.5	62.5±19.2	15.0±13.4	1.12 (0.84–1.47)	0.47 (0.32–0.60)
LVSV by volume (mL)	72	54.9±18.4	70.1±21.9	15.1±13.8	1.10 (0.83–1.43)	0.45 (0.30–0.58)
LVSVI (mL·m^−2^)	67	27.6±8.1	36.2±9.5	8.6±7.7	1.11 (0.83–1.45)	0.53 (0.38–0.65)
LVEDV (mL)	72	87.4±29.1	105.0±35.1	17.7±18.5	0.96 (0.70–1.27)	0.35 (0.20–0.49)
LVESV (mL)	72	32.4±15.8	35.0±18.6	2.6±10.2	0.25 (0.02–0.50)	NTE
LVEF^#^ (%)	72	63.8±10.6	67.7±9.2	3.9±7.8	0.50 (0.27–0.76)	0.20 (0.04–0.35)
LVCO^+^ (L·min^−1^)	64	3.5±1.1	4.3±1.4	0.8±0.9	0.96 (0.69–1.29)	0.42 (0.26–0.55)
LVCI^+^ (L·min^−1^·m^−2^)	64	2.0±0.6	2.5±0.7	0.5±0.5	0.96 (0.69–1.30)	0.43 (0.27–0.57)
LV mass (g)	72	104.4±25.8	108.8±27.5	4.4±11.2	0.39 (0.16–0.65)	NTE
RVEDV/LVEDV	72	0.5±0.3	0.3±0.3	−0.2±0.3	0.92 (0.67–1.23)	0.43 (0.27–0.56)
RVESV/LVESV	72	1.0±0.5	0.7±0.5	−0.3±0.5	0.72 (0.48–1.01)	0.39 (0.23–0.53)
**Functional and other non-invasive variables**						
6MWD (m)	76	406.8±118.4	450.8±120.3	44.0±81.6	0.54 (0.31–0.80)	0.28 (0.12–0.42)
Absolute NT-proBNP (ng·L^−1^)	68	1210.9±2019.2	441.6±933.4	−769.4±1514.5	0.51 (0.27–0.78)	0.42 (0.27–0.56)
Log-transformed NT-proBNP (ng·L^−1^)	68	6.2±1.3	5.3±1.1	−0.9±1.0	0.94 (0.70–1.18)	0.42 (0.27–0.56)
Borg dyspnoea score	70	3.5±2.0	3.1±2.0	−0.5±2.1	0.22 (−0.01–0.47)	0.20 (0.04–0.35)
DBP (mmHg)	78	73.7±11.5	69.6±10.8	−4.1±9.5	0.43 (0.21–0.68)	0.21 (0.05–0.35)
SBP (mmHg)	78	116.3±13.8	114.5±13.4	−1.7±12.9	NTE	NTE
Heart rate (beats·min^−1^)	78	79.7±13.0	76.2±13.3	−3.5±13.0	0.27 (0.05–0.50)	0.19 (0.03–0.34)

Baseline, week 52 and change from baseline to week 52 data are presented as mean±sd. ^#^: determined from standard volumetric measurements; ^¶^: determined from pulmonary artery flow; ^+^: determined from aortic flow. Due to rounding, in some cases the difference between week 52 and baseline values does not equate to the change in baseline value. For the standardised treatment effect sizes: dark blue indicates a large change, mid-blue indicates a medium change and light blue indicates a small/fair change; NTE indicates no treatment effect (Cohen's d <0.20; Cliff's delta <0.11). CL: confidence limit; RV: right ventricular; RVSV: RV stroke volume; RVSVI: RVSV index; RVEDV: RV end-diastolic volume; RVESV: RV end-systolic volume; RVEF: RV ejection fraction; RVCO: RV cardiac output; RVCI: RV cardiac index; LV: left ventricular; LVSV: LV stroke volume; LVSVI: LVSV index; LVEDV: LV end-diastolic volume; LVESV: LV end-systolic volume; LVEF: LV ejection fraction; LVCO: LV cardiac output; LVCI: LV cardiac index; 6MWD: 6-min walk distance; NT-proBNP: N-terminal pro-brain natriuretic peptide; DBP: diastolic blood pressure; SBP: systolic blood pressure.

**FIGURE 3:**
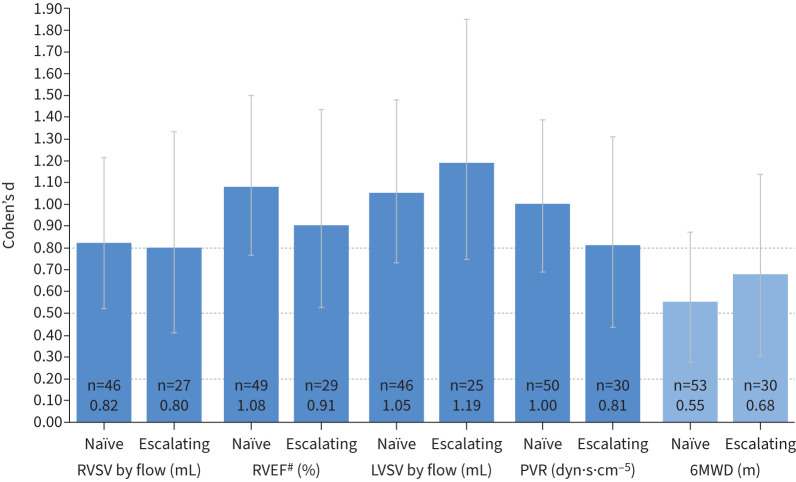
Cohen's d statistics for cardiac magnetic resonance imaging (cMRI), right heart catheterisation (RHC) and functional variables in treatment-naïve (n=56) and escalating (n=31) patients at week 26. ^#^: determined from standard volumetric measurements. n: number of patients with baseline and post-baseline measurements for a specific variable; Cohen's d statistics are reported below the n-values. For the standardised treatment effect sizes (STES): dark blue indicates a large change and mid-blue indicates a medium change. The dotted lines at 0.20, 0.50 and 0.80 indicate the thresholds for a fair, medium or large change in STES, respectively. RV: right ventricular; RVSV: RV stroke volume; RVEF: RV ejection fraction; LVSV: left ventricular stroke volume; PVR: pulmonary vascular resistance; 6MWD: 6-min walk distance.

For all variables measured, Cohen's d STES observed at week 52 were similar to the week 26 values (tables 2 and 3 and figures 1 and 2). The STES observed at week 26 for cMRI variables that are considered prognostic in PAH (RVSV by flow, RVEF and LVSV by flow [11, 20, 21]) or that are currently accepted PAH clinical trial end-points (PVR and 6MWD [3]) were similar between treatment-naïve patients and patients escalating therapy (figure 3). These variables met the Cohen's d threshold for large STES in both treatment-naïve patients and patients escalating therapy, with the exception of 6MWD, for which medium STES were observed for both subgroups. Week 52 data are shown in supplementary figure S3a.

### Supportive analysis of STES by Cliff's delta statistic

Cliff's delta statistics for cMRI, RHC, functional and other non-invasive variables at weeks 26 and 52 are shown in tables 2 and 3 and supplementary figures S2b, S3b, S3c and S4. For all variables, the pattern of STES was broadly similar to that of the Cohen's d statistic.

## Discussion

In this *post hoc* analysis of REPAIR, the Cohen's d STES of macitentan (alone or in combination with a PDE5i) were large for half of the cMRI metrics measured, similar to those observed for PVR and log-transformed NT-proBNP, and greater than for 6MWD. This included the parameters RVSV, LVSV and RVEF, which are prognostic in PAH [9, 21–23]. Changes were consistent at weeks 26 and 52, in treatment-naïve patients and patients escalating therapy, and using both the Cohen's d and Cliff's delta tests. The non-invasive nature of cMRI, its high repeatability and ability to reflect disease severity and predict clinically relevant outcomes, as well as the sensitivity of cMRI-assessed variables to macitentan and other PAH therapies [9, 13, 21–24], position cMRI-assessed variables as ideal parameters to assess treatment response for future PAH trials.

RHC is the gold standard for the measurement of PAP and its measurements are predictive of survival in PAH [25]. RHC-derived PVR is a commonly used PAH end-point and had a large Cohen's d treatment effect size at week 26 following macitentan treatment in our study. Other purportedly prognostic RHC parameters including stroke volume, mPAP and cardiac index also had large STES at week 26; however, their predictive status has been inconsistently reported and requires further investigation [26, 27]. Additionally, RHC is an invasive procedure and may not be a practical follow-up approach for some patients [28].

In this analysis the currently accepted non-invasive end-point used by regulatory authorities, 6MWD [3], had a moderate Cohen's d treatment effect size at week 26, consistent with results obtained using the non-parametric Cliff's delta method. However, potential outliers at baseline resulted in large variability and a skewed distribution of the data [16], which may have impacted the estimates. Furthermore, the relatively high 6MWD at baseline (mean of 401 m) potentially limited the amount patients could improve, reducing the likelihood of observing a clinically relevant change in 6MWD compared with other measures. Several other limitations of 6MWD have been highlighted in the literature, and include a minimal or lack of association with other short- and long-term outcomes and difficulty detecting treatment effects of add-on or sequential therapy [2].

NT-proBNP is another non-invasive, sensitive measure of RV function and has been shown to be prognostic in PAH [28–30], although it is not specific to the disease and its potential to be used as a surrogate end-point for long-term outcomes in PAH has yet to be validated [31]. At week 26, a fair Cohen's d STES was observed for absolute NT-proBNP; however, this increased to a large effect upon log-transformation of the data; the effect for Cliff's delta estimate was medium in both cases. These results are in contrast to those observed in a previous study [13], where the STES of log-transformed NT-proBNP was only fair; however, this could be a reflection of the previous study's smaller sample size. The prognostic utility of log-transformed NT-proBNP has been highlighted in an earlier study [32] and the consistency of the Cliff's delta analyses indicates a better performance in estimating the STES of log-transformed NT-proBNP. Overall, the observed discrepancies suggest that caution must be taken when choosing the method of STES derivation for variables that are non-normally distributed.

There are several reasons to consider cMRI metrics when assessing the potential of new non-invasive end-points in PAH. Previous studies have demonstrated cMRI measures of RV and LV structure and function to be prognostic in PAH [9, 11, 20–23], and able to reveal disease progression prior to clinical deterioration (worsening of functional class and decreases in 6MWD) [9, 10]. RV measures of structure include RV mass and RVESV, and of function include cardiac index, RVSV and RVEF [16], while corresponding measures of the left heart provide complimentary information on LV structure and function. In this study, half of the cMRI variables measured, including RVSV by flow, RVEF and LVEDV, all of which are strongly prognostic in PAH [11, 20, 21], had large Cohen's d STES in patients receiving macitentan alone, or in combination with a PDE5i at week 26. These results are comparable to those observed for PVR, mPAP, cardiac index and stroke volume measured by RHC, and greater than those observed for 6MWD and absolute NT-proBNP levels. Overall, these results are in agreement with a previous study that observed a large treatment effect size for RVEF, a medium treatment effect size for RVSV and a fair treatment effect size for 6MWD in PAH patients initiating or escalating PAH therapy [13]. Of note, LVSV was the only parameter in this analysis for which the lower limit of the Cohen's d 95% CL remained above the 0.80 threshold at both weeks 26 and 52. LVSV is more accurate and reproducible to measure by cMRI than RVSV [33] and is prognostic in PAH [34]. This, combined with the fact that for many PAH patients LVSV reflects more accurately the output of the heart [35], makes this parameter a particularly attractive candidate for further investigation as a surrogate end-point.

In addition to REPAIR, several studies have reported that cMRI-derived variables can be used to monitor the impact of PAH therapies [12, 23, 24], aid risk stratification in PAH [11] and identify patients with maladaptive remodelling at high risk to treatment failure [36]. Treatment effect is often highest during the first therapeutic regimen (monotherapy or combination therapy) and the magnitude of improvement for many variables decreases with the introduction of additional therapy. This has been clearly demonstrated with the 6MWD, where a “ceiling effect” has been observed for patients with high baseline values and for those on background therapy who have already improved their exercise capacity [37]. For an end-point to be most useful, it is important that the variable has a robust STES in both initial and escalation treatment settings. In our study, observed treatment effect sizes of cMRI variables in treatment-naïve patients and patients escalating therapy were similar, suggesting that like RHC-derived PVR, cMRI variables are able to detect treatment effect sizes in patients receiving PAH background therapy.

The robust STES analysis presented here on the REPAIR dataset [16] adds to the body of evidence suggesting that non-invasive cMRI metrics, reflective of the underlying disease process and related to long-term clinical outcomes, warrant investigation as surrogate end-points in PAH trials. However, as for RVSV [38–40], a clinically relevant minimally important difference (MID) must be determined for each cMRI metric in order to confirm its suitability as a surrogate end-point. One recent study using change in WHO FC as an anchor determined an 11% change in RVEDV to be clinically relevant [38]. Another study calculated the MID in cMRI metrics using two distribution-based methods and two anchor-based measures benchmarked to how a patient “feels” (emPHasis-10 quality of life questionnaire), “functions” (incremental shuttle walk test) and “survives” (1-year mortality post-follow-up) to identify clinically relevant thresholds for change [40]. The authors of the study reported a MID of 5% absolute change for RVEF and of −17 mL and +10 mL for RVESV or RVEDV for improvement and worsening, respectively [40]. Reassuringly, in this analysis of the REPAIR safety set, the observed improvements in RVEF (+9.1%) and RVESV (−18.2 mL) at week 26 were above these thresholds. The improvement for RVEDV (5.2% change; −7.8 mL) did not reach this threshold, which may reflect the less severe disease status of the patients in the REPAIR study. The discrepancy between improvement in RVESV and RVEDV is also reflected in the Cohen's d estimates (0.78 and 0.28, respectively) and may suggest that RVESV is the more robust metric to assess therapy response.

There are several limitations to these analyses. First, this is a *post hoc* analysis and thus susceptible to potential bias (no adjustments for multiplicity, patients may not have had complete data for all variables). Second, in the REPAIR study there were very few patients with disease progression, thus it was not possible to determine whether the cMRI variables measured also reflect disease worsening. Third, these data reflect the treatment effect of macitentan either alone or in combination with a PDE5i. Although a recently published study examining non-invasive end-points in PAH in a smaller cohort of patients has shown similar findings [13], further studies are needed to determine if the results are applicable to other PAH therapies. Finally, the published thresholds for interpreting Cohen's d and Cliff's delta estimates have not been validated in PAH. In the REPAIR study, RVSV was chosen as the cMRI end-point based on the availability of a published MID (8–12 mL) [39], while PVR was chosen as it is an established end-point in PAH [3]. In this analysis, the Cohen's d estimate was ≥0.80 for both primary end-points. The fact that the changes in the two primary end-points were both significant and clinically relevant suggests that the published thresholds of ≥0.80 for Cohen's d are appropriate in the context of PAH to show a large treatment effect size. The thresholds for the Cliff's delta statistic [19] are less established than those of Cohen's d [17]; however, the results using this approach were largely in line with the Cohen's d estimates, suggesting that the Cliff's delta thresholds may also be appropriate in the PAH setting.

### Conclusions

In the REPAIR study, sensitivity to the treatment effect of macitentan, alone or as part of combination therapy with a PDE5i, was comparable for several cMRI and haemodynamic variables with known prognostic value in PAH, and greater than that of 6MWD in patients with PAH. The STES in this analysis were consistent at weeks 26 and 52, and between treatment-naïve patients and patients escalating therapy. Which cMRI metric or combination of metrics to use, how best to standardise the measurements and whether particular metrics are sensitive to different types of therapeutic interventions requires further evaluation, but the results of this *post hoc* analysis suggest that cMRI variables should be investigated as surrogate end-points in PAH trials.

## Supplementary material

10.1183/23120541.00547-2023.Supp1 **Please note:** supplementary material is not edited by the Editorial Office, and is uploaded as it has been supplied by the author.
Supplementary material 00547-2023.SUPPLEMENT
