# Supplementary material for: Comparison of cardiac magnetic resonance imaging, functional and haemodynamic variables in pulmonary arterial hypertension: insights from REPAIR
Source: ERJ Open Res. 2024 Feb 12;10(1):00547-2023. doi: 10.1183/23120541.00547-2023 (PMC10860210; doi:10.1183/23120541.00547-2023)
Supplement: Supplementary file 1 [file 00547-2023.SUPPLEMENT.pdf]

Comparison of cardiac magnetic resonance imaging, functional and haemodynamic variables clinical endpoints in pulmonary arterial hypertension: insights from the REPAIR study

Supplemental Material

Authors: David G Kiely[1,2], Richard Channick[3], Dayana Flores[4], Nazzareno Galiè[5], Gwen MacDonald[4], J Tim Marcus[6], Lada Mitchell[4], Andrew Peacock[7], Stephan Rosenkranz[8], Ahmed Tawakol[9], Adam Torbicki[10], Anton Vonk Noordegraaf[6], Andrew J Swift[2]

1 SUPPLEMENTAL MATERIAL

Supplemental Methods

Statistical analysis

Cohen’s d treatment effect size estimator

Cohen's d effect size is computed according to the formula:[1]

$$\text{Cohen's d}=\frac{Mean\ of\ the\ change\ from\ baseline\ to\ Week\ 26\ or\ Week\ 52}{Standard\ Deviation\ of\ the\ change\ from\ baseline\ to\ Week\ 26\ or\ Week\ 52}$$

Cliff's Delta analysis (non-parametric)

The Cliff’s Delta estimator is obtained according to the formula:[2]

Variables for which a post-baseline measurement greater than the baseline measurement denotes improvement:

$$Delta = \frac{\#(\mathrm{X}_1 > \mathrm{X}_2) - \#(\mathrm{X}_1 < \mathrm{X}_2)}{\mathrm{n}_1\mathrm{n}_2}$$

Variables for which a post-baseline measurement less than the baseline measurement denotes improvement:

$$Delta = \frac{\#(\mathrm{X}_2 > \mathrm{X}_1) - \#(\mathrm{X}_2 < \mathrm{X}_1)}{\mathrm{n}_1\mathrm{n}_2}$$

Where for each variable of interest:

- X_1 is the post-baseline measurement
- X_2 is the baseline measurement
- n_1 is the number of patients with a post-baseline measurement
- n_2 is the number of patients with a baseline measurement

Supplemental Appendix I

Institutional Review Boards/ethics committees

Country	Site #	Name of IEC/IRB
**Malaysia**	9001	Local: IJN Research Ethics Committee
**Singapore**	1201	Local: NHG Domain Specific Review Board
	1202*	Local: NHG Domain Specific Review Board
**Hong Kong**	1301	Local: Institutional Review Board of the University of Hong Kong/ Hospital Authority Hong Kong West Cluster
	1302	Local: Institutional Review Board of the University of Hong Kong/ Hospital Authority Hong Kong West Cluster
	1303	Local: Research Ethics Committee (Kowloon Central/Kowloon East Cluster)
**Russia**	1101	Local: Ethics Committee of Federal State Budgetary Institution “Almazov National Medical Research Centre” of the Ministry of Health of the Russian Federation.
	1102	Local: Independent Ethics committee of clinical trials of Federal State Budgetary Institution “National Medical Research Centre of Cardiology” of the Ministry of Health of the Russian Federation.
**Israel**	8001	Local: Ethics Helsinki Committee
	8002	Local: Ethics Helsinki Committee
**United States of America**	1001*	Local: Partners Human Research Committee
	1002*	Local: Western Institutional Review Board
	1004*	Local: Western Institutional Review Board
	1005	Central: Western Institutional Review Board
	1006	Local: UT Southwestern Institutional Review Board
	1007*	Local: Aurora Health Care Research Subject Protection Program and IRB office

	1008*	Local: Weill Cornell Medicine IRB
	1009*	Local: University of Minnesota Human Research Protection Program
	1010*	Local: Western Institutional Review Board
	1012	Local: The Washington University in St Louis IRB
	1014*	Local: Western Institutional Review Board
	1015*	Local: Houston Methodist Research Institute IRB
**Australia**	2001*	Central: The Prince Charles Hospital HREC
**France**	3002, 3003*, 3004*, 3005, 3007, 3008, 3010*, 3011	Central: Comité de Protection des Personnes Est III
**Germany**	4001, 4002*, 4003, 4004*, 4005, 4006*, 4007, 4008*	Central: Ethic Committee of the Medical Faculty of Heidelberg
**Italy**	5001*	Central: Comitato Etico Azienda Ospedaliero Universitaria di Bologna Policlinico S.Orsola-Malpighi
	5002	Local: Comitato di Bioetica della Fondazione IRCCS Policlinico S. Matteo di Pavia
**Netherlands**	6001, 6002, 6004, 6005*, 6006*	Central: Medical Ethical Committee VU
**United Kingdom**	7001*, 7003, 7005	Central: NRES Committee Yorkshire & The Humber – Leeds West Health Research Authority

*Sites that were initiated but that did not enrol any subjects.

Supplemental Figure 1. Study design

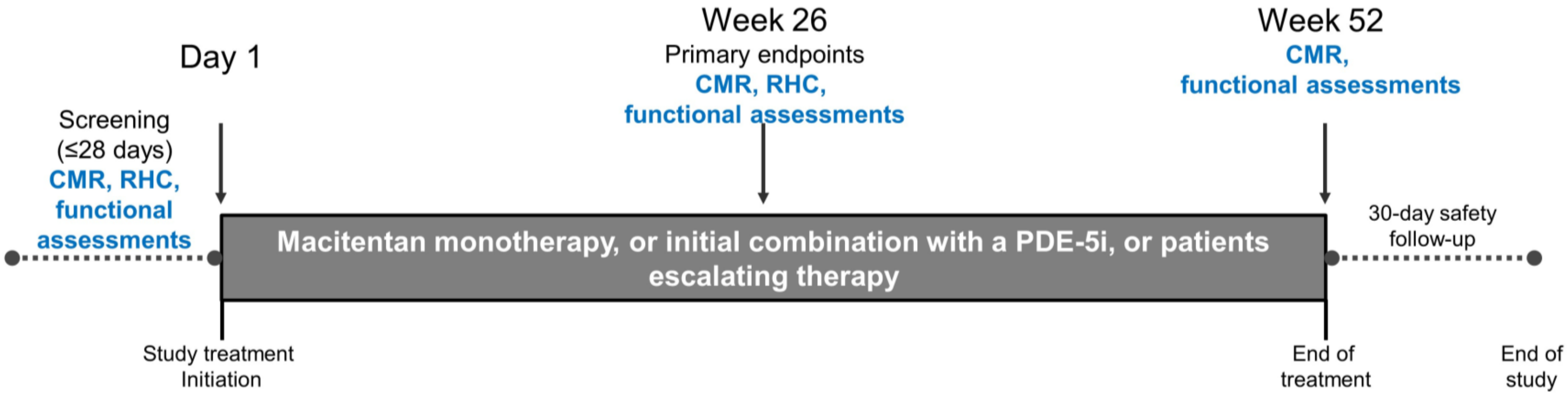

Study design figure has been previously published.[3] CMR: cardiac magnetic resonance imaging; PDE-5i: phosphodiesterase 5 inhibitor; RHC: right heart catheterisation.

Supplemental Figure 2. Cohen's d (a) and Cliff's delta (b) statistics for absolute and log-transformed NT-proBNP (ng/L) values at Weeks 26 and 52 (safety set)

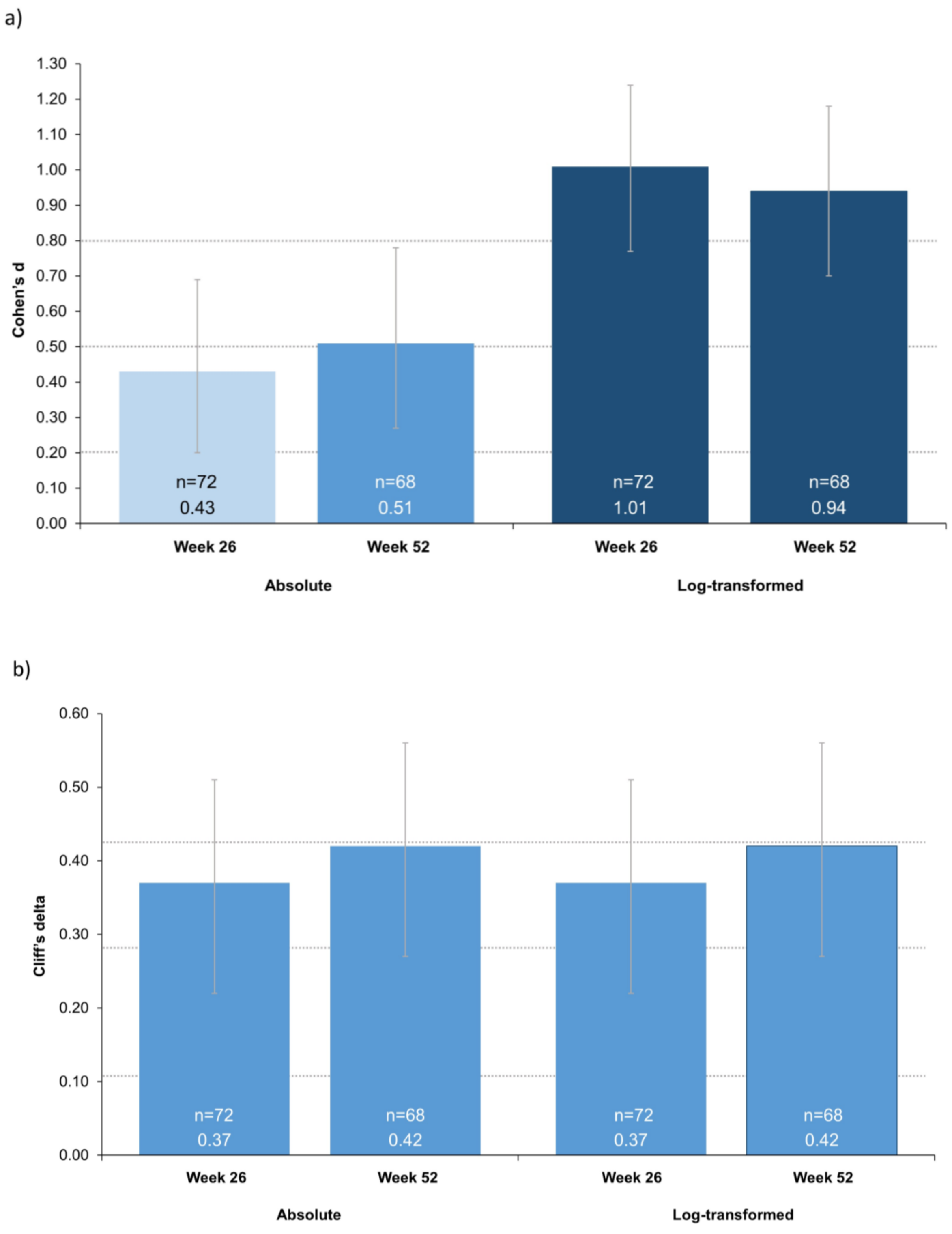

n = number of patients with baseline and post-baseline measurements for a specific variable; Cohen's d and Cliff's delta statistics are reported below the n values. For the standardised treatment effect sizes, dark blue indicates a large change, mid-blue indicates a medium change, light blue indicates a small/fair change. NT-proBNP: N-terminal pro-brain natriuretic peptide.

Supplemental Figure 3. (a) Cohen's d statistic at Week 52, and Cliff's delta statistic at (b) Week 26, and (c) at Week 52 for CMR and functional variables in treatment-naïve (N = 56) and escalating (N = 31) patients (safety set)

a)

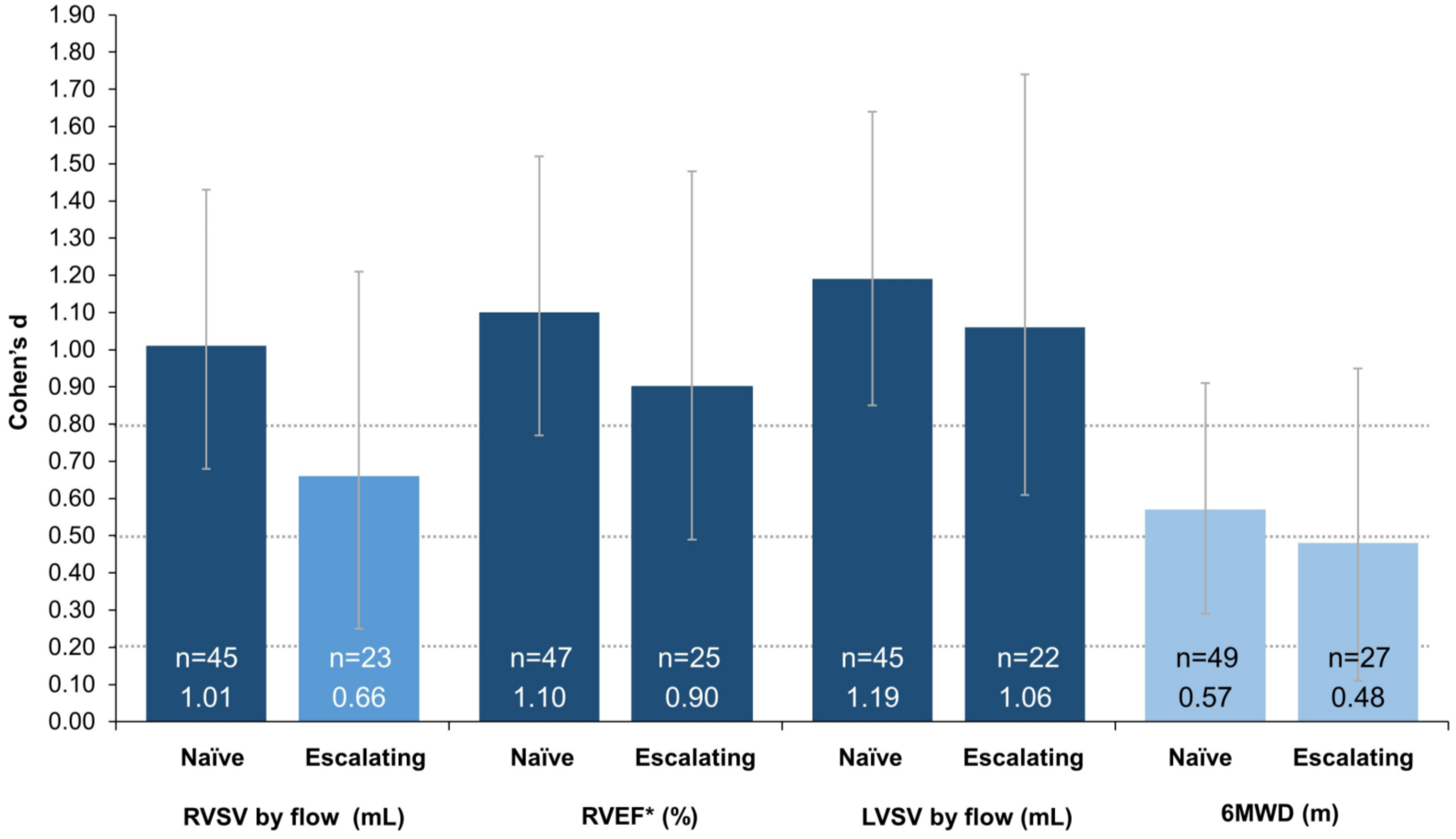

b)

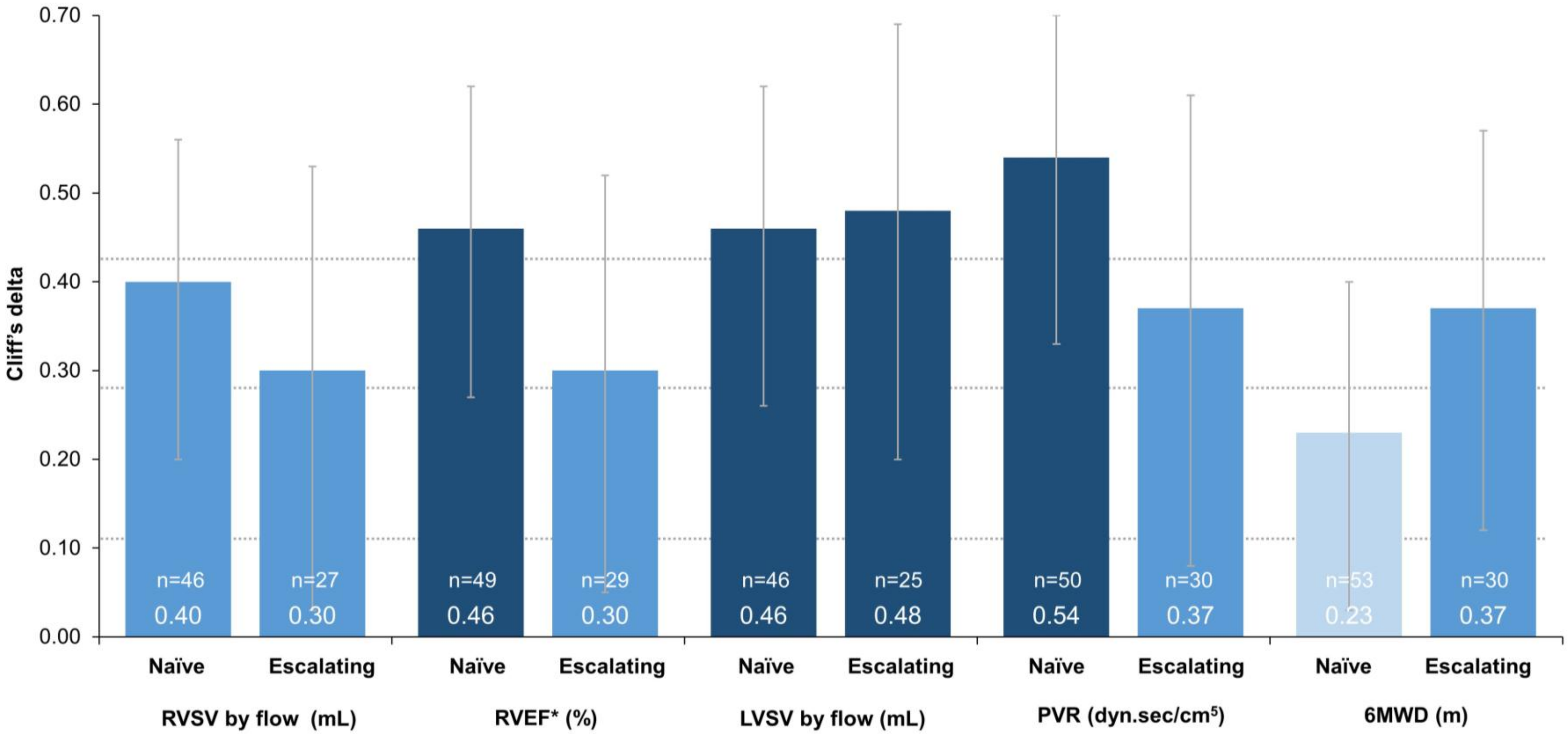
Cliff's delta
0.70
0.60
0.50
0.40
0.30
0.20
0.10
0.00
n=46
0.40
n=27
0.30
n=49
0.46
n=29
0.30
n=46
0.46
n=25
0.48
n=50
0.54
n=30
0.37
n=53
0.23
n=30
0.37
Naïve
Escalating
Naïve
Escalating
Naïve
Escalating
Naïve
Escalating
Naïve
Escalating
RVSV by flow (mL)
RVEF* (%)
LVSV by flow (mL)
PVR (dyn.sec/cm5)
6MWD (m)

c)

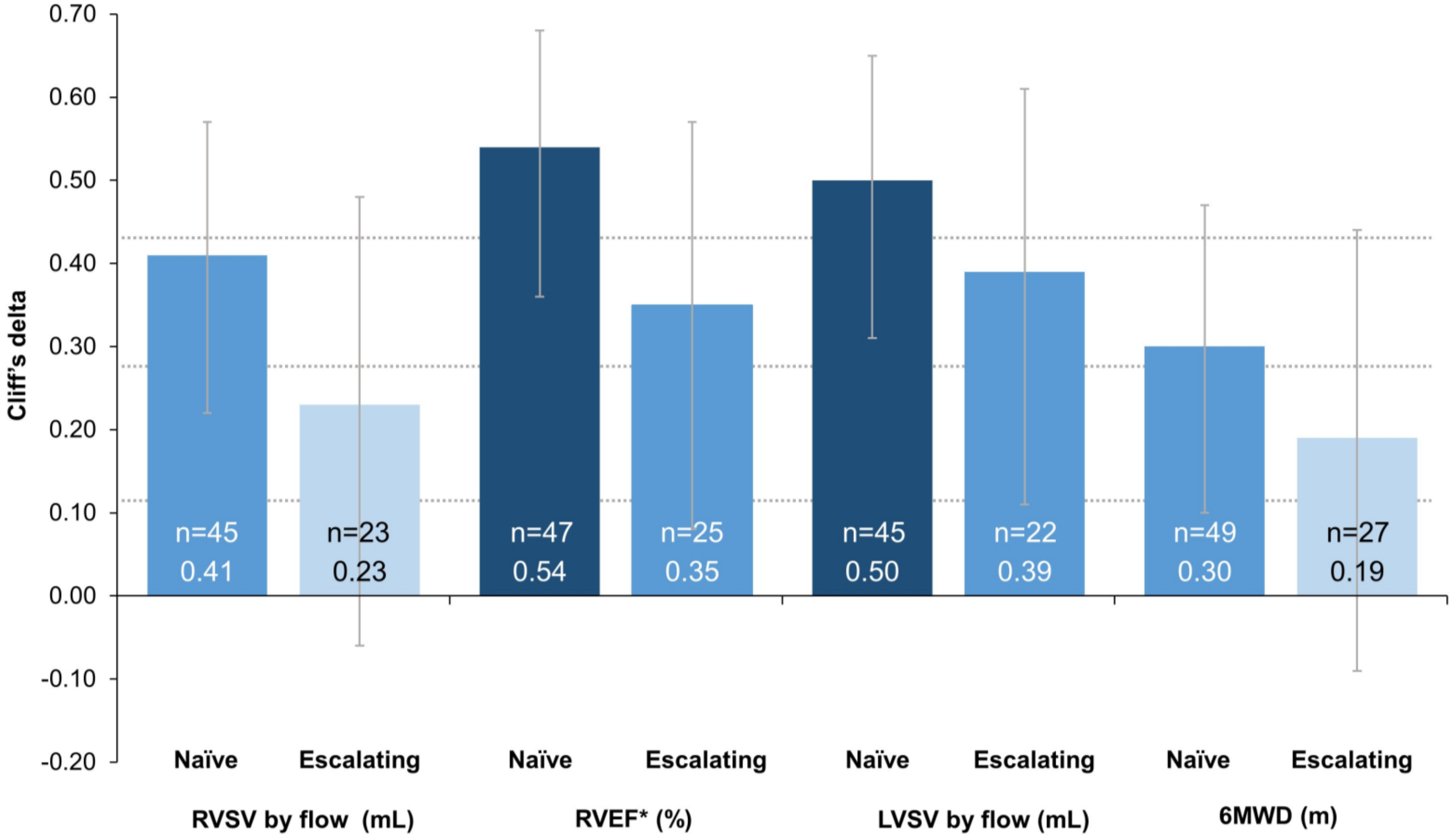

*Determined from standard volumetric measurements. n = number of patients with baseline and post-baseline measurements for a specific variable; Cohen's d and Cliff's delta statistic are reported below the n values. For the standardised treatment effect sizes, dark blue indicates a large change, mid-blue indicates a medium change, light blue indicates a small/fair change. 6MWD: 6-minute walk distance; CMR: cardiac magnetic resonance imaging; LVSV: left ventricular stroke volume; PVR: pulmonary vascular resistance; RVEF: right ventricular ejection fraction; RVSV: right ventricular stroke volume.

Supplemental Figure 4. Cliff's delta statistics at (a) Week 26 and (b) Week 52 for CMR and functional and other non-invasive variables (safety set)

a)

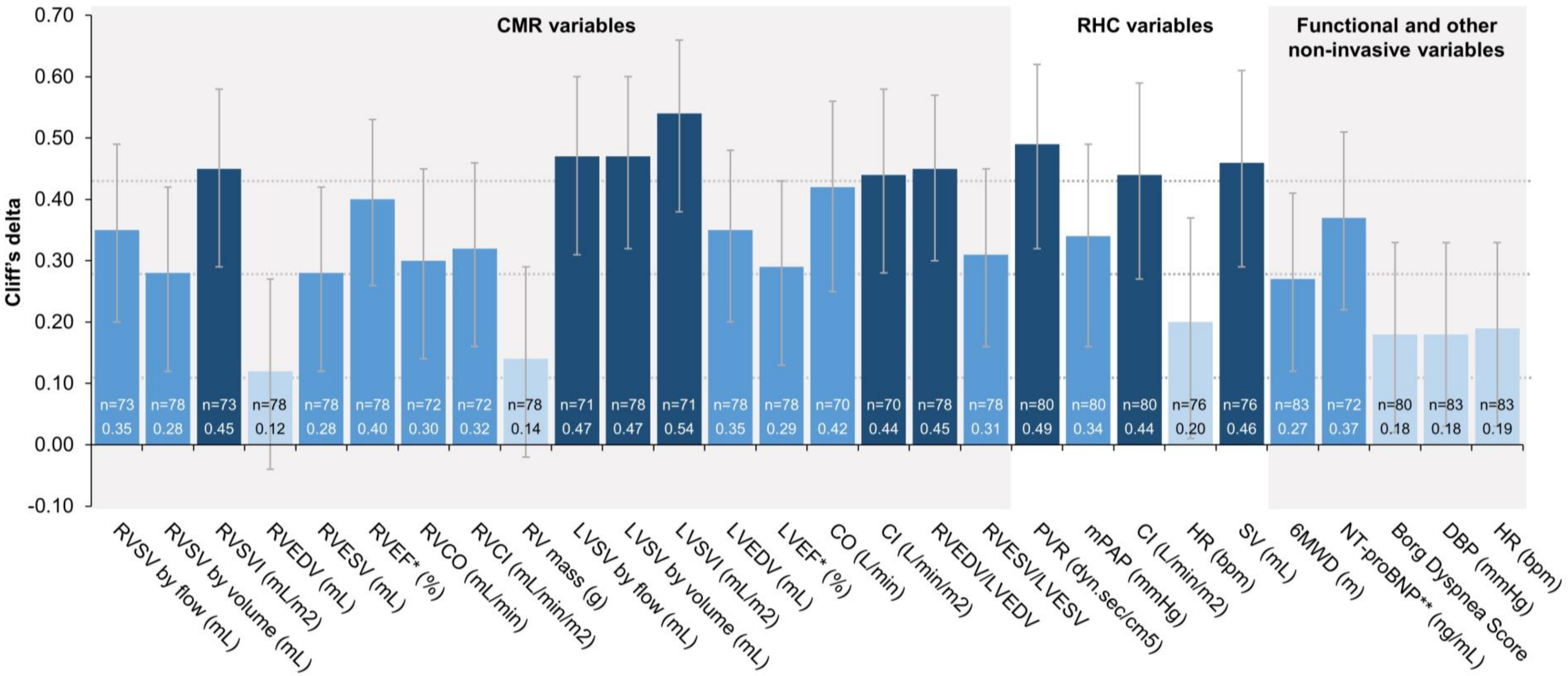

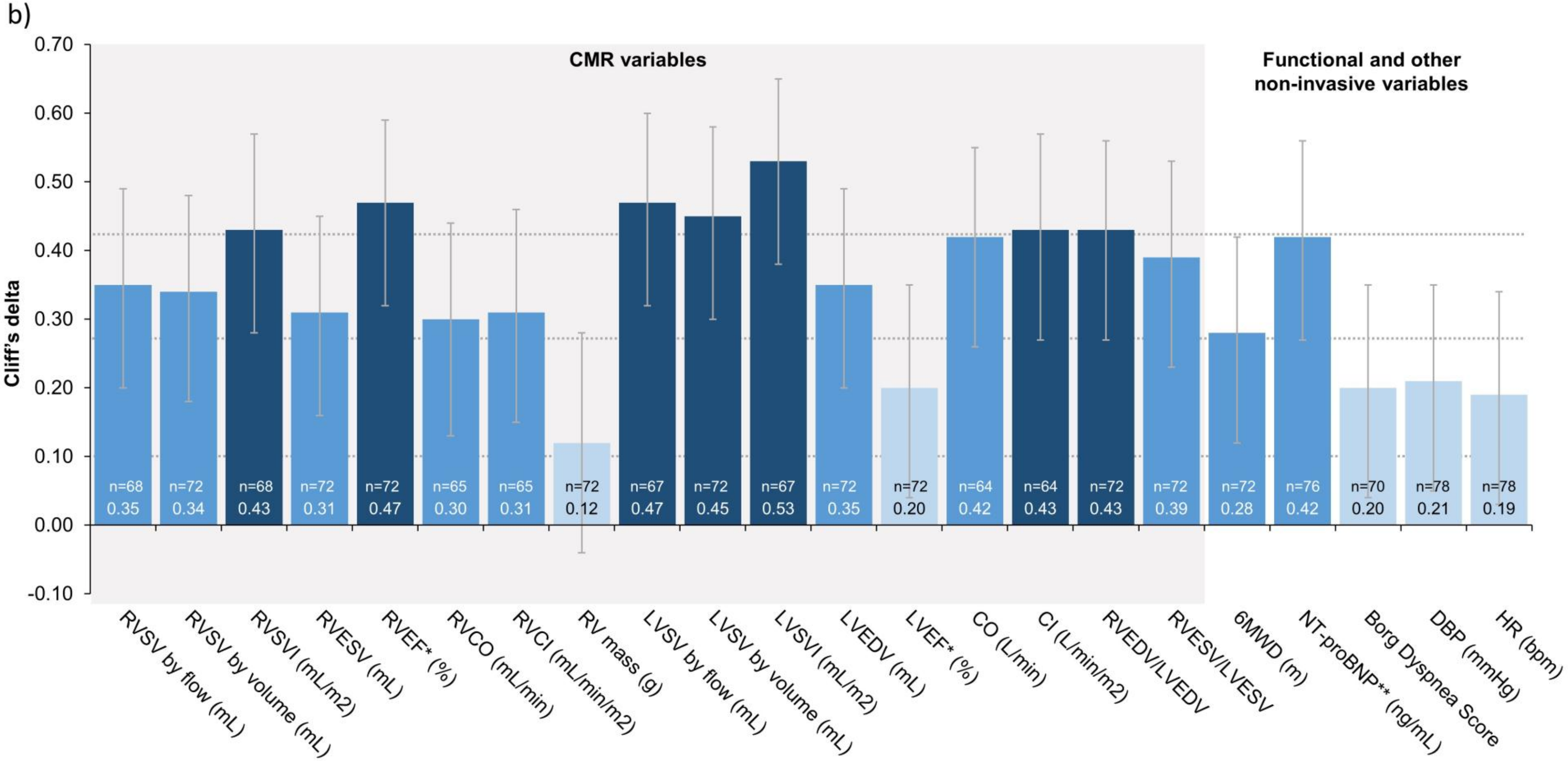

*Determined from standard volumetric measurements. **Log-transformed. Variables with no treatment effect are not included. n = number of patients with baseline and post-baseline measurements for a specific variable; Cliff's delta statistics are reported below the n values. For the standardised treatment effect sizes, dark blue indicates a large change, mid-blue indicates a medium change, light blue indicates a small/fair change. 6MWD: 6-minute walk distance; CI: cardiac index; CMR: cardiac magnetic resonance imaging; CO: cardiac output; DBP: diastolic blood pressure; HR: heart rate; LV: left ventricular; LVEDV: LV end-diastolic volume; LVEF: LV ejection fraction; LVESV: LV end-systolic volume; LVSV: LV stroke volume; LVSVI: LVSV index; mPAP: mean pulmonary artery pressure; NT-proBNP: N-terminal pro-brain natriuretic peptide; PVR: pulmonary vascular resistance; RHC: right heart catheterisation; RV: right ventricular; RVCI: RV cardiac index; RVCO: RV cardiac output; RVEDV: RV end-diastolic volume; RVEF: RV ejection fraction; RVESV: RV end-systolic volume; RVSV: RV stroke volume; RVSVI: RVSV index; SV: stroke volume

References

1 Fitts DA. Commentary on “A review of effect sizes and their confidence intervals, Part I: The Cohen’s d family”: The degrees of freedom for paired samples designs. *TQMP* 2020; 16: 281–294.

2 Macbeth G, Razumiejczyk E, Ledesma RD. Cliff’s Delta Calculator: A non-parametric effect size program for two groups of observations. *Universitas Psychologica* 2011; 10: 545–555.

3 Vonk Noordegraaf A, Channick R, Cottreel E, et al. The REPAIR Study: Effects of Macitentan on RV Structure and Function in Pulmonary Arterial Hypertension. *JACC Cardiovasc Imaging* 2022; 15: 240–253.
